# Change in healthcare resource use and associated costs of patients with metastatic lung cancer between 2013 and 2021: an observational study from the French national health data system

**DOI:** 10.1007/s10198-025-01864-6

**Published:** 2025-11-12

**Authors:** Christos Chouaid, Clarisse Marchal, Marion Apert, Lionel Bensimon, Valérie Guimard, Mélanie Née, Manon Belhassen, Gérard de Pouvourville, Jean-Yves Blay

**Affiliations:** 1 Service de Pneumologie, Centre Hospitalier Intercommunal de Créteil, Créteil, France; 2 PELyon, Lyon, France; 3 MSD France, Puteaux, France; 4 ESSEC, Cergy-Pontoise, France; 5 Centre Léon Bérard, Lyon, France

**Keywords:** Metastatic lung cancer, Healthcare resource utilization (HCRU), Costs, Claims database, France

## Abstract

**Introduction:**

Treatment landscape in metastatic lung cancer has progressed quickly over the last decade, mainly due to immunotherapies and targeted therapies. This study aimed to describe changes in costs associated with metastatic lung cancer in France.

**Materials and methods:**

A cohort of patients identified between 2013 and 2021 with lung cancer and a marker of metastases (ICD-10 code or reimbursement for Bevacizumab or Pemetrexed) was built from the French claims database. Healthcare resource use was described each year. The trend in total mean monthly costs (MMC) expressed in gross price over the study period was studied using Joinpoint software.

**Results:**

Between 2013 and 2021, 147,760 metastatic lung cancer patients were identified (men :66.5%, median age: 66 years). The annual cost for all patients increased from 611,074,408€ in 2013 (*N* = 24,595) to 1,308,745,922€ in 2021 (*N* = 40,321). The MMC per patient decreased from 2013 to 2015 (from 5,853€ to 4,895€), by 9.37% per year (95%CI: -17.30; -0.69), then stabilized (0.90%; 95%CI: -0.84; 2.66). An increase in drug acquisition MMC in the same proportions as the decrease in full hospitalization MMC was observed, excluding 2020.

**Conclusion:**

Although the global cost of metastatic lung cancer management has increased, the MMC per patient has not notably surged between 2013 and 2021. Drug acquisition MMC increase was offset by reduced full hospitalization MMC (excluding 2020), resulting in no significant uptrend. These results suggest that the global management cost increase is mainly driven by epidemiological and demographic factors. This highlights the importance of continued investment in prevention and in therapeutic innovations that may improve outcomes.

**Supplementary Information:**

The online version contains supplementary material available at 10.1007/s10198-025-01864-6.

## Introduction

In France, lung cancer is the second most common cancer in men and the third in women, with 52,777 new cases in 2023, and the leading cause of cancer-related deaths [1, 2]. Over the 2010–2023 period, the number of newly-diagnosed patients fell slightly in men (−0.5% per year) and rose in women (+ 4.3% per year) [1]. According to the French KBP-2020-CPHG study, 60.4% of lung cancer patients were diagnosed at stage IV [3]. Moreover, among cancers diagnosed at a localized or locally advanced stage, about 40% will progress to an advanced or metastatic stage within a year [4]. Lung cancer is therefore associated with a poor prognosis and represents a major public health issue.

For many years, the management of advanced or metastatic lung cancer was a primary systemic anti-cancer therapy based on platinum (since the 70 s) with or without an association to a third-generation cytotoxic agent (gemcitabine, vinorelbine and taxanes) (since the 90 s). In 2004, EGFR mutation was discovered, followed, a few years after, by the identification of other oncogenic alterations (e.g. ALK, MET, KRAS, ROS-1, RET, BRAF, NTRK). These recent breakthroughs in precision medicine have had an important impact on overall survival, safety and quality of life of patients with first the development of targeted therapy (since 2006 in non-mutated cancer and since 2010 in EGFR positive cancer) [5, 6]. More recently, the arrival of PD-1/PD-L1 checkpoint inhibitors has brought new hope in the management of this disease (under an early access program for nivolumab since July 2015, then May 2017 for pembrolizumab, and February 2019 for atezolizumab), and became the new standard of care in advanced lung disease.

Since then, the rapid evolution of treatment options in metastatic lung cancer and its clinical benefits have been associated with economic concerns from various stakeholders including payers, health care professionals, policymakers, and patients. Several French public reports have highlighted the increasing acquisition costs of these treatments and the associated impact on public health insurance expenses [7–9]. In recent years, multiple medico-economic evaluations have been conducted by the French health technology assessment body (CEESP, part of HAS) regarding PD-1/PD-L1 inhibitors, as well as targeted therapies, in the treatment of lung cancer. While some incremental cost-effectiveness ratios (ICERs) were deemed acceptable, others were considered high or very high by CEESP, reflecting differences in clinical benefit, target population, and treatment setting. These disparities highlight the importance of further investigating their broader impact on healthcare budgets, beyond individual cost-effectiveness metrics.

However, since the introduction of immunotherapies, only a few international studies have described cost trends in metastatic lung cancer [10, 11] and none have been conducted in France. The evolution of global expenditures in the country — beyond drug acquisition alone — remains insufficiently documented.

The goal of this study was to provide with real world evidence public health information to help address affordability concerns from stakeholders about innovation in oncology, in metastatic lung cancer. For this purpose, the temporal changes of the burden of disease were described in the French nation-wide claims database.

## Materials and methods

### Data source

This study was conducted from the French national health data system (Système National des Données de Santé, [SNDS]) which contains anonymous individual information about primary and secondary care utilization and currently covers 98.8% of population leaving in France (about 67 million in 2021). Data recorded include patients’ general characteristics, non-hospital reimbursed healthcare expenditures (visits and medical procedures, lab tests, drugs, and medical devices), and all hospital discharge summaries, including associated costs [12]. This database can provide a comprehensive estimate of the burden of diseases. Hospital medications are included in the cost of the stay, except for costly or innovative drugs which are eligible for separate funding.

### Objectives

The study objectives were to describe change in annual Health Care Resource Utilization (HCRU) and associated costs of metastatic lung cancer management between 2013 and 2021.

### Study population and study design

This study is an observational, retrospective, dynamic cohort of patients identified with a metastatic lung cancer between January 1 st, 2013 and December 31 st, 2021 in France. Patients were included if they met the two following criteria during the inclusion period:

1. A Long-Term Disease status (LTD, i.e. status providing full coverage for all medical expenses related to a specific condition) or hospitalization for lung cancer (C34 or C399 ICD-10 codes),
2. A LTD or hospitalization for secondary malignant neoplasm (C77-C79 ICD-10 codes) or at least one reimbursement for bevacizumab or pemetrexed, which were used as proxies for metastatic disease (bevacizumab and pemetrexed were used as proxies for metastatic disease, given their specific use at this stage during the study period. This helped identify patients without a recorded metastasis code).

The first occurrence of one of these inclusion criteria was defined as the index date. Patients were followed until death, the last patient’s health record (i.e. last care recorded in the database before a period of 6 months without any reimbursed care), or the end of the study period (i.e. December 31 st, 2021), whichever occurred first. A pre-study period of seven years before index date was used to select only patients with primary lung cancer by excluding patients with probable pulmonary metastases (i.e. patients with a marker of metastases or of another cancer before the first lung cancer diagnosis). Patients were excluded if they were under 18 years old at index date or if they were not continuously covered by the general health insurance scheme during the 7 years preceding the index date and throughout the follow-up period, to ensure completeness of healthcare data.

### Variables

Baseline characteristics (age, sex, comorbidities, Charlson score) of the global cohort were described. HCRU and associated costs were described each calendar year between 2013 and 2021, according to the following care items: anti-cancer therapy (immunotherapy, targeted therapy, costly or innovative chemotherapy which are not included in the in-patient cost, monoclonal antibody) (see Suppl Table 1), other medications, day and full hospitalizations, home hospitalizations, rehabilitation cares, drug infusion (hospitalization for anti-cancer therapy infusion), medical visits (general practitioners, office-based specialists, and all hospital-based physicians), outpatient medical procedures (imaging, surgeries and all technical acts), lab tests, sick leaves, and medical transports. The number of patients with a disability pension was also estimated. Costs were estimated from a health insurance perspective (i.e., total reimbursed amount). All costs were adjusted for inflation using the consumer price index (CPI) for health care services published by the French National Institute of Statistics and Economic Studies (INSEE), and expressed in 2021 euros. This approach allows for a consistent comparison of expenditures over time, providing a more accurate description of spending trends from the payer’s perspective.

### Statistical analyses

For each year and each category of care, we described both the number of patients with at least one reimbursement (i.e., users) and the mean number of care units—such as dispensings for treatments, hospital stays, or medical procedures—calculated among users and across the entire study population (including patients with no reimbursement for the given care, i.e., zero values). Sick leaves and disability pensions were also described for patients aged 62 years or less (i.e. working population defined according to INSEE data) [13]. The monthly cost per patient was computed overall and for each healthcare expenditure item. These analyses were also carried out by sex and age groups (< 55, [55–70[, [70–80[, ≥ 80) during the calendar year. Lastly, the global cost of all patients with metastatic lung cancer each year was estimated.

The trend in the overall mean monthly cost over the 2013–2021 period was studied using the National Cancer Institute’s (NCI) Joinpoint Regression Program (Statistical Methodology and Applications Branch, Surveillance Research Program, National Cancer Institute, Bethesda, MD, USA), version 5.4.0 [14]. The analysis started with the minimum number of joinpoints (i.e., 0 joinpoint representing a straight line) and we tested whether one joinpoint was statistically significant and could be added to the model (one joinpoint being the maximum number recommended with seven observation points). Costs trend was measured using the average annual percent change (AAPC) over the study period and the annual percentage change (APC).

All other statistical analyses were performed using SAS Enterprise Guide (SAS Institute Inc., Cary, North Carolina, USA) version 8.3 update 7.

### Ethics

This study was approved by the French Institute for Health Data (approval no. 2262161 from 3 September 2020). It was conducted with anonymized data, as requested by the National Informatics and Liberty Commission [CNIL], approval no.920444), from 4 March 2021.

## Results

### Selection and follow-up

**Fig. 1:**
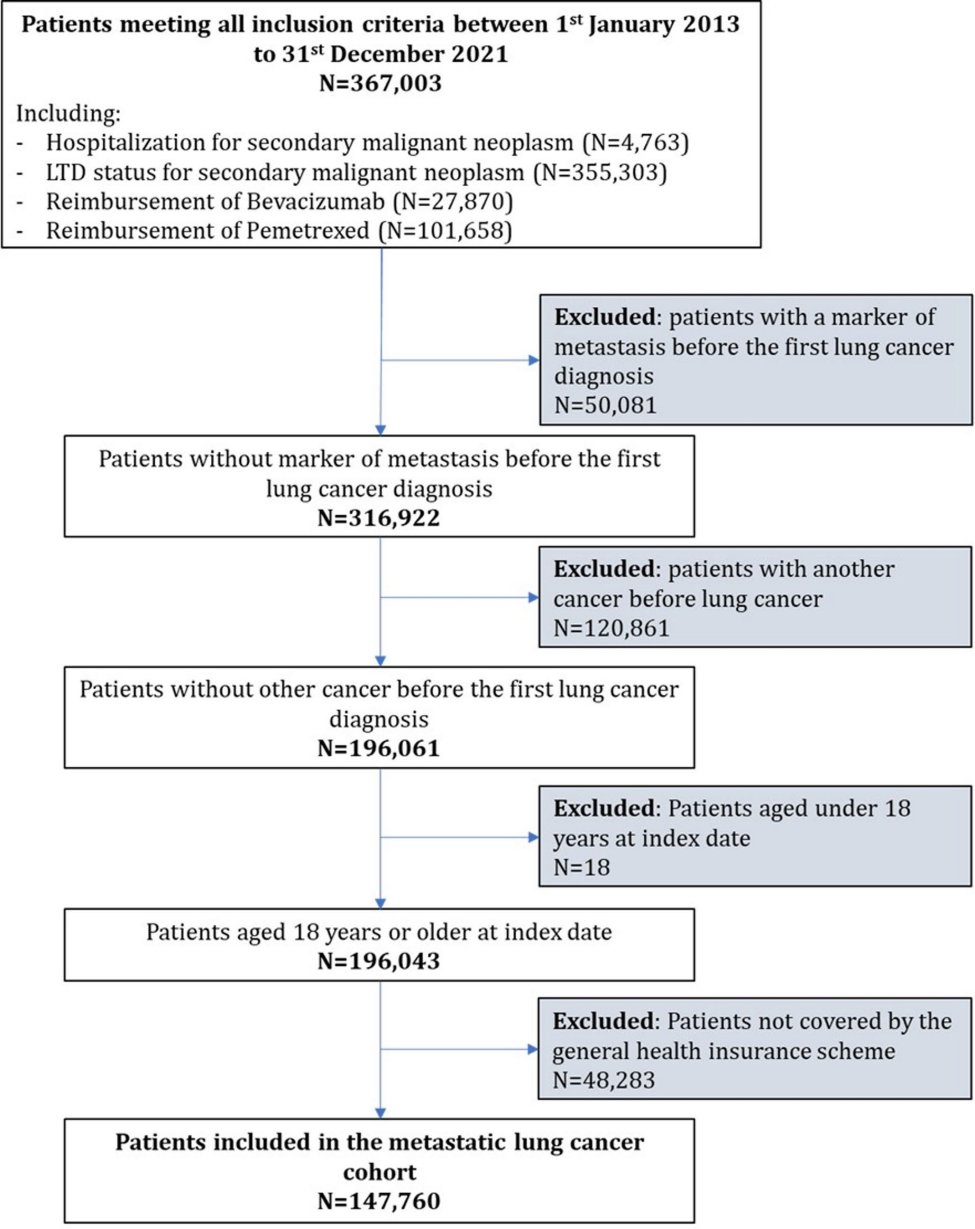
Flow chart

Between 2013 and 2021, both lung cancer diagnosis and metastasis marker were recorded in 367,003 patients (Fig. 1). After excluding patients with a marker of metastases or of another cancer before the first lung cancer diagnosis (i.e. probable pulmonary metastases) (*N* = 170,942), patients under 18 (*N* = 18) and those not covered by the general health insurance scheme (*N* = 48,283), 147,760 patients were included in the metastatic lung cancer cohort. The number of patients living with a metastatic lung cancer almost doubled over the study period, from 24,595 in 2013 to 40,321 in 2021, including 14,587 (59.3%) and 15,225 (37.8%) newly diagnosed patients, respectively. The median follow-up was 6.8 months (Q1-Q3: 2.3–17.6).

### Patients’ characteristics

Two thirds of patients were male (66.5%; Table 1). Median age was 66.0 years (Q1-Q3: 58.0–73.0). Patients had a high comorbidity burden (median Charlson score of 14.0 (Q1-Q3: 13.0–15.0)), including 54.9% with cardiovascular diseases, 27.9% with severe chronic respiratory insufficiency and 16.7% with diabetes.

**Table 1:** Characteristics of the study population

		Metastatic lung cancer cohort (*N* = 147,760)
Sex, *n*(%)		
Male		98,221 (66.5%)
Age (years)		
Median (Q1 - Q3)		66.0 (58.0–73.0)
Age (years), n(%)		
< 55		21,637 (14.6%)
[55–70[		72,762 (49.2%)
[70–80[		34,932 (23.6%)
≥ 80		18,429 (12.5%)
Comorbidities		
Diabetes type 1 and 2		24,627 (16.7%)
Cardiovasculardiseases		81,125 (54.9%)
Inflammatorydiseases		3,071 (2.1%)
Severechronicrespiratoryinsufficiency		41,238 (27.9%)
Severe chronic nephropathy and nephrotic syndrome		6,403 (4.3%)
Cancers other than lung cancer		25,360 (17.2%)
Malnutrition		53,356 (36.1%)
Long term oxygenotherapy use, n(%)		11,399 (7.7%)
Charlson score		
Median (Q1 - Q3)		14.0 (13.0–15.0)

*For the population of less than 65 years

### Healthcare Resource Use

**Fig. 2:**
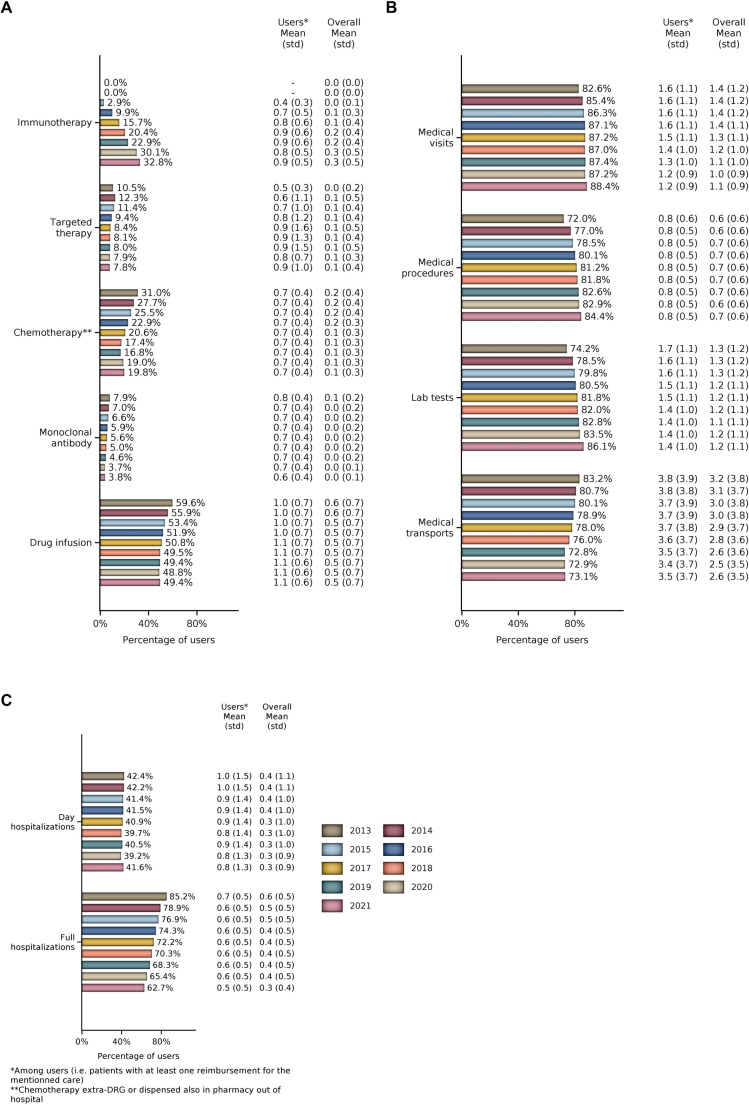
Percentage of patients with at least one care and mean monthly number of care, in the overall cohort and in users, by health expenditure item: **a**) cancer treatments **b**) outpatient cares **c**) hospitalizations

The percentage of patients receiving at least one administration of immunotherapy (all treatment lines considered) increased from 2015 (2.9%) to 2021 (32.8%) (Fig. 2). Conversely, a decrease in the percentage of patients receiving other monoclonal antibodies (from 7.9% in 2013 to 3.8% in 2021) and targeted therapies (from 12.3% in 2014 to 8.0% in 2019) was observed. The use of costly or innovative chemotherapies also decreased from 31.0% in 2013 to 16.8% in 2019 but increased in 2020 and 2021 (19.0% and 19.8%, respectively). While the use of day and home hospitalizations were stable (about 40% and 7% of patients each year, respectively), the percentage of patients with full hospitalizations considerably decreased over the study period (from 85.2% to 62.7%), as well as those experiencing rehabilitation care (from 13.8% to 8.9%). Conversely, the percentage of patients with outpatient care increased over the study period: medical visits (from 82.6% to 88.4%), lab tests (from 74.2% to 86.1%), medical procedures (from 72.0% to 84.4%), other medications (from 82.5% to 90.0%). Among users, the mean number of medical visits per month has dropped (from 1.6 in 2016 to 1.2 in 2021), as has the mean number of lab tests (from 1.7 in 2013 to 1.4 in 2021). Patients had in average about one medical procedure (0.8 in 2021) and two dispensings of anti-cancer drugs (2.2 in 2021) per month, all types of anti-cancer agents combined (e.g., chemotherapy, targeted therapy, immunotherapy), without distinction between treatment combinations. The percentage of patients aged 62 or less benefiting from sick leaves decreased from 44.2% in 2013 to 32.6% in 2021, while the percentage of patients receiving disability pension increased from 12.9% to 18.4% in 2019, then stabilized.

### Costs

#### Overall cohort

**Fig. 3:**
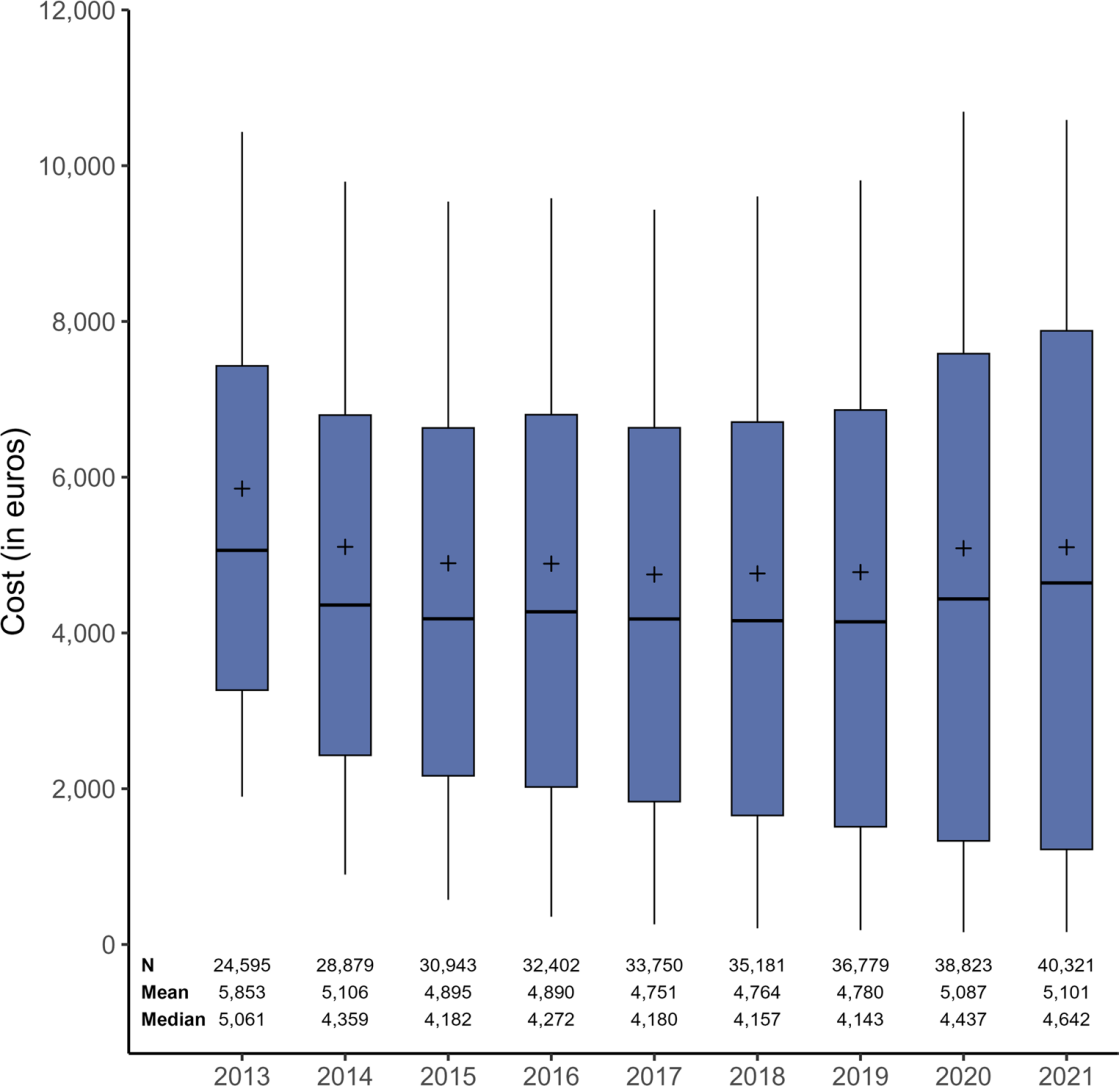
Boxplot of mean monthly costs per patients living with a metastatic lung cancer between 2013 and 2021. Whiskers represent the 10th and 90th percentiles (P10–P90)

**Fig. 4:**
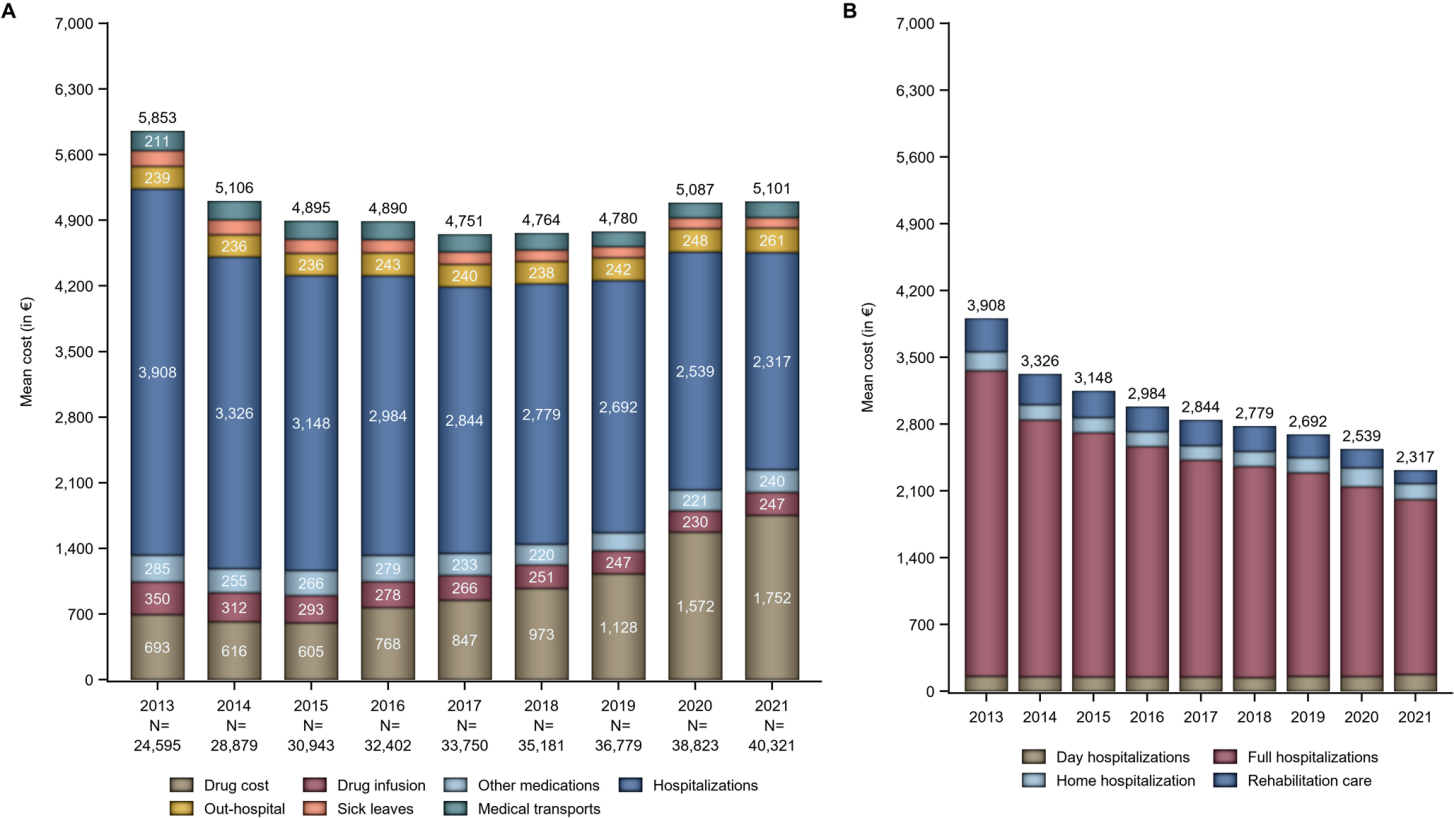
Breakdown of mean monthly costs per patient by health expenditure item: **a**) overall **b**) cost by type of hospitalization

The global cost of patients living with metastatic lung cancer each year has more than doubled over the 2013–2021 period, from 611,074,408€ in 2013 (*N* = 24,595) to 1,308,745,922€ in 2021 (*N* = 40,321). A decrease in the mean monthly cost per patient from 5,853€ in 2013 to 4,895€ in 2015 was observed (Fig. 3). This result was mainly driven by the decrease in full hospitalizations costs from 3,204€ (54.7% of total cost) to 2,562€ per month (52.3% of total cost) (Fig. 4). Between 2015 and 2021, anti-cancer therapy acquisition costs (i.e. drug costs and infusion-related hospitalizations) increased by + 123% (from €898 to €1,999 per patient per month), while overall hospitalization costs decreased by − 26% (from €3,148 to €2,317 per patient per month). Only in 2020 did the rise in acquisition costs (427€) exceed the decrease in hospitalization costs (153€), resulting in an increase in average monthly costs to 5,087€. The increase in anti-cancer therapy acquisition costs was mainly driven by immunotherapy costs (from 38€ per month in 2015 to 1,371€ in 2021) and targeted therapies costs (from 113€ per month in 2013 to 278€ in 2021).

#### Subgroups

The mean monthly costs declined from 2013 to 2015 (to 2017 for patients under 55 years old), then stabilized, in both men (from 5,892€ to 4,993€ in 2015) and women (from 5,771€ to 4,704€ in 2015) (see Suppl Fig. 1), and in every age group (< 55 years old: from 6,272€ to 5,319€ in 2015; [55–70[: from 5,859€ to 4,841€; [70–80[: 5,531€ to 4,665€; ≥ 80 years old: 5,785€ to 4,917€) (see Suppl Fig. 2). An increase in the mean monthly cost in 2020 was also observed for each of these subgroups, except for patients aged over 80 years for whom the cost remained stable.

#### Trend analysis

**Fig. 5:**
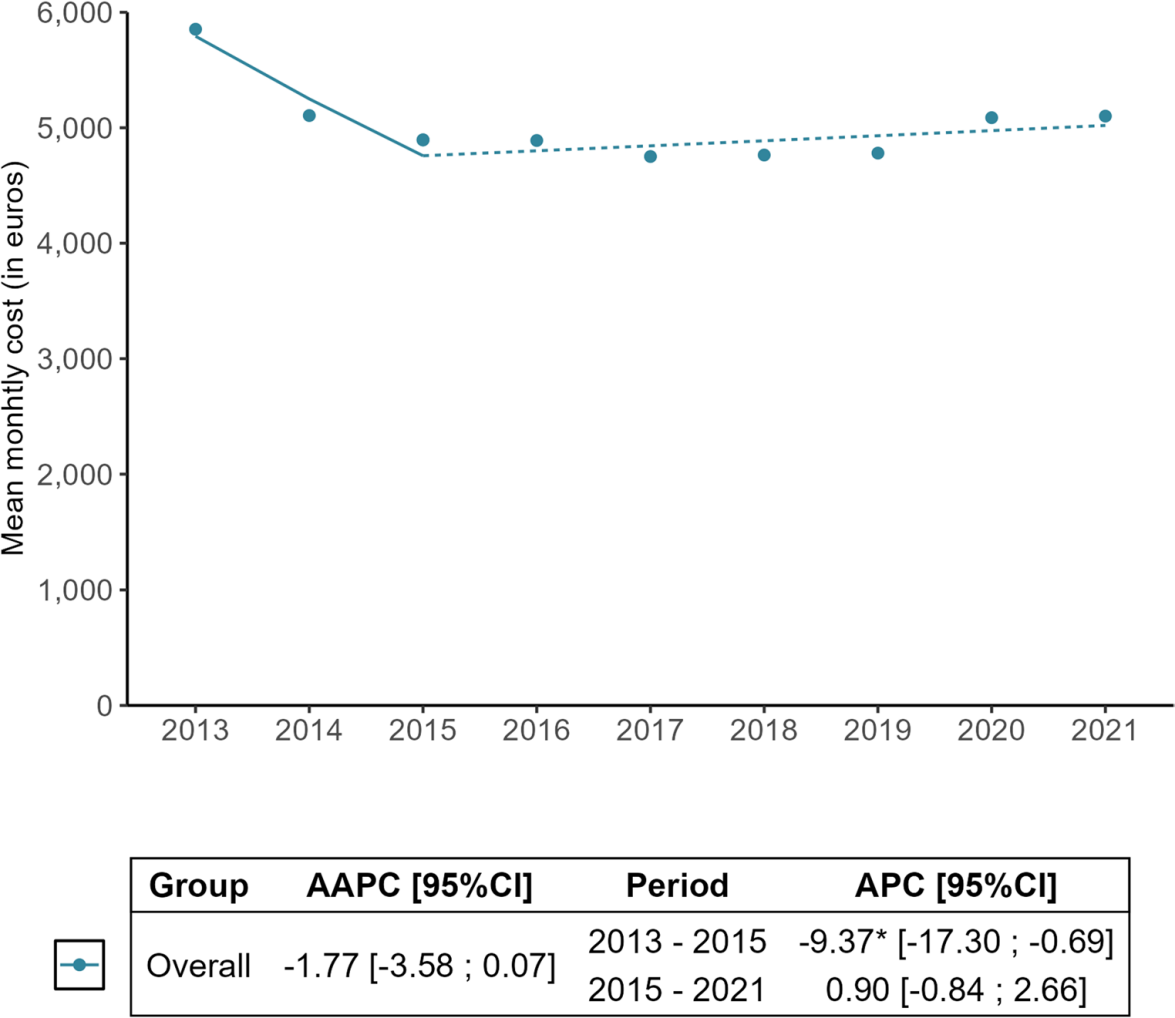
Trend in the mean monthly cost per patient with metastatic lung cancer from a health insurance perspective between 2013 and 2021

From 2013 to 2015, a statistically significant decrease of 9.37% per year (95% CI: −17.30 to −0.69, *p* < 0.0001) (Fig. 5) was observed. The overall mean monthly cost then stabilized between 2015 and 2021 (annual percentage change of 0.90%; 95% CI: −0.84 to 2.66).

## Discussion

To our knowledge, this study is the first to provide data on HCRU and overall costs for patients with metastatic lung cancer over time in France, which is of interest within a period of unprecedented improvements in survival rates for patients with metastatic lung cancer in recent years. These results are of particular interest as lung cancer is the second most costly cancer, after breast cancer; is mainly diagnosed at metastatic settings and it has been an area of many innovations in the last decade [15, 16]. Although the global cost of metastatic lung cancer management has increased, the mean monthly cost per patient has not notably surged between 2013 and 2021. Drug acquisition cost was offset by reduced full hospitalization cost (excluding 2020), resulting in no significant uptrend.

In France, the National Health Service (NHS – Caisse Nationale de l’Assurance Maladie), which publishes reimbursed expenditure by pathology each year, confirmed that those allocated to lung cancer management (all stages combined) in the active treatment phase increased every year [16]. Although these estimates are reported for all stages (about 2.3 billion euros in France in 2019), it is interesting to put them into perspective with our results reporting data specifically for metastatic patients. The increase observed in the overall cost in our study could be explained by the demographic change (population increase and aging), the increased risk of cancer, and the improvement of life expectancy of patient treated for a metastatic lung cancer. When put into perspective with available literature, in 2019, the mean monthly cost per patient at metastatic stage estimated in this study was 2.5 times higher than those estimated by the NHS for overall patients, whatever the stage, in the active treatment phase (€57,360/year versus €23,100/year), which is consistent with the fact that lung cancer management costs increase with stage of the disease [17, 18]. NHS has also observed a decrease in hospitalization costs and an increase in costly drug costs between 2015 and 2019 [16], although, contrary to what has been showed in this study at metastatic stage, hospitalization costs did not offset drug costs for patients at any stage. It can be assumed that the evolution of the treatment landscape has had a greater impact on the most severe patients, at metastatic stage, whose needs for hospitalization may have been further reduced than those of less severe patients. The few studies abroad that have investigated the economic burden of lung cancer since the evolution of the treatment landscape have observed increasing cost for systemic therapies [10, 11, 19]. If the study of Zhang et al. showed that inpatient, outpatient and pharmacy costs remained relatively stable [11], Korytowsky and al., who matched two cohorts before and after approval of immunotherapy, showed lower rates of emergency department visits and hospitalizations, and thus lower associated costs, in the post-immunotherapy period, favorably affecting the per patient per month total cost of care ($12,681 versus $10,758, over the pre- and post-immunotherapy periods, respectively) [19].

Over the last decade, the emergence of new therapies, such as immunotherapy and targeted therapies, has marked a significant shift in the treatment of metastatic lung cancer, bringing promising advances that may have contributed to a reduction in full hospitalizations. Compared with conventional treatments, these innovative therapies offer better symptom management, limiting severe complications, and reducing the need for frequent full hospitalization. Moreover, in a context marked by an aging population, the development of chronic pathologies and a strong aspiration to home care, France requires an in-depth restructuring of the organization of its health services. One of the main strategies which was chosen is the development of ambulatory care, initiated as early as the 2000 s, but which really emerged in 2013 in a report published by the French Court of Auditors, and which was reinforced by the 2015–2017 three-year plan implemented in 2014 and by the French healthcare system modernization law of January 26, 2016 [20–22]. The drop in mean monthly costs observed between 2013 and 2014 could therefore be the result of the implementation of these health policies. One of the two main aims of this plan is to reduce the number of full hospitalizations (as observed in this study) in favor of day hospitalizations. As no ambulatory surgery was expected in this population (no tumor surgery performed at metastatic stage), the stable use of day hospitalizations described in this study is consistent. Instead, there seems to have been a transfer to outpatient care.

The potential contribution of heath policies implemented in France since 2013 on the modification of healthcare resource utilization have, however, been impacted by the covid 19 pandemic in 2020. This is the only year for which there has been a slight increase in the mean monthly cost per patient in this study. COVID-19 pandemic particularly impacted lung cancer patients, who had the highest risk of severe COVID-19 illness and mortality (25–30%), after patients treated for hematological malignancies [23]. Many learned medical societies and cooperative groups have rapidly issued recommendations for the management and prevention of COVID-19 in patients with lung cancer [24–26–27

The SNDS is a national healthcare claims database which covers more than 98% of the French population. Overall, this database is representative of the full population leaving in France. In addition, it contains comprehensive information about treatments and use of reimbursed healthcare resources, as well as exhaustive costs. All costs in our study were expressed in inflation-adjusted euros, allowing for consistent comparisons over time. This has enabled us to provide the most comprehensive view of the burden of metastatic lung cancer for the French NHS among the available data sources.

Focusing on non-small cell lung cancer was not possible because there is no specific ICD-10 code or treatment allowing us to distinguish these two types of lung cancer. Nevertheless, small cell lung cancer only representing 15% of lung cancers, the bias can be considered as limited. Moreover, the coding of metastases is not mandatory and can be delayed or not provided, which may have led to a selection bias if the coding depends on patient severity. Indeed, it seems that metastases coding is carried out more frequently in full hospitalizations (to increase payment to the stay), than in the case of day hospitalization for treatment infusions. This means that some of the less severe patients, who only come to hospital for treatment infusion, may have been missed, leading to an overestimation of the costs. However, given the severity of metastatic lung cancer, this bias is certainly limited. Moreover, bevacizumab and pemetrexed administrations, two treatments specific used in the metastatic stage, were also used as proxy to reduce this bias. In any cases, this bias was similar each year and have no impact on trend tests. Lastly, by using only hospital discharge diagnoses and hospital treatments as selection criteria, it is likely that part of patients receiving only outpatient treatments have been missed, leading again to a potential selection bias towards the most severe patients. Although it probably concerns only a small proportion of our population, the burden described in this study are probably not generalizable to the entire population of patients with metastatic lung cancer, but more likely only to the most severe patients. Furthermore, our results also reflect French healthcare policies and are therefore difficult to generalize to metastatic lung cancer patients worldwide. However, our results seem to be in line with those of other studies abroad [10, 19].

In addition, primary cancer localization is unfortunately not available in the SNDS database. Thus, we developed an algorithm, based on a pre-study period of seven years, to identify lung cancer and to exclude patients with pulmonary metastases of another cancer. This algorithm was chosen to be specific, which probably led to the exclusion of some patients who had metastatic lung cancer, but which enabled us to describe HCRU and cost estimates specific to metastatic lung cancer.

Finally, inpatient reimbursements for conventional chemotherapy (i.e. drugs that are neither costly nor innovative) are not available in the SNDS database, as they are directly included in the cost of the hospital stay. This did not allow us to describe the overall use of chemotherapies, but this had no impact on the mean monthly costs per patient and per year. However, it should be noted that for all anti-cancer drugs, acquisition costs were estimated based on a gross price and not the net ones negotiated with Economic Committee. This leads to an overestimation of acquisition costs in our analysis, especially as antineoplastic agents have the highest average rebate rate per therapeutic class (34.5% according to the latest 2022 activity report published by the Economic Committee). Lastly, the non-inclusion of a part of patients only treated with outpatient treatments may have led to an underestimation of the use of targeted therapies and may explain the decline in the use of targeted therapies: since patients treated only with outpatient treatments at the start of the study period have more follow-up than patients included in 2020 or 2021, they are more likely to be hospitalized or receive inpatient treatment due to disease progression, and therefore more likely to be included in this study. Further investigations are needed to investigate if the addition of patient treated solely with outpatient treatment will confirm this point and the trends observed in this study.

## Conclusion

To conclude, although the treatment landscape of metastatic lung cancer management has rapidly evolved over the last decade and is associated with improved survival, the mean monthly cost of management per patient has not notably surged between 2013 and 2021. It should be noted that these trends are based on a drug acquisition cost expressed in gross price and not the net ones negotiated with Economic Committee, which may lead to an overestimation of acquisition costs in our analysis. In addition, all costs were expressed in inflation-adjusted euros, allowing for accurate comparisons over time. Considering these learnings, it can be assumed that the rise in the overall cost of managing patients living with a metastatic lung cancer in France between 2013 and 2021, has mainly been driven by demographic changes, an increased incidence of cancer, and by an improved patient survival, highlighting the crucial importance of continued investments in both prevention and innovation. Further investigations are necessary to validate the initial trends with longer follow-up. 

## Supplementary Information

Below is the link to the electronic supplementary material.

Supplementary Material 1 (DOCX 177 KB)

## Data Availability

Due to NHS and SNDS rules, no data sharing is possible as access to data is restricted to habilitated and qualified researchers (Mélanie Née is habilitated and qualified).
