# Supplementary material for: Change in healthcare resource use and associated costs of patients with metastatic lung cancer between 2013 and 2021: an observational study from the French national health data system
Source: Eur J Health Econ. 2025 Nov 12;27(4):881–91. doi: 10.1007/s10198-025-01864-6 (PMC13350122; doi:10.1007/s10198-025-01864-6)
Supplement: Supplementary file 1 — Supplementary Material 1 (DOCX 177 KB) [file 10198_2025_1864_MOESM1_ESM.docx]

**Supplementary material**

Suppl Table 1: Systemic anticancer therapy of interest

| Treatment class | Product |
| --- | --- |
| Chemotherapy | Carboplatine  Cisplatine  Docetaxel  Gemcitabine  Paclitaxel  Pemetrexed*  Vinorelbine** |
| Targeted therapy | Afatinib**  Alectinib**  Brigatinib*  Ceritinib**  Crizotinib**  Erlotinib**  Gefitinib**  Lorlatinib**  Osimertinib** |
| Immunotherapy | Atezolizumab*  Nivolumab*  Pembrolizumab* |
| Monoclonal antibody | Bevacizumab* |

Treatments still under personal early access “ATUn” cannot be studied.

* Treatment extra DRG

** Dispensed also in pharmacy out of hospital


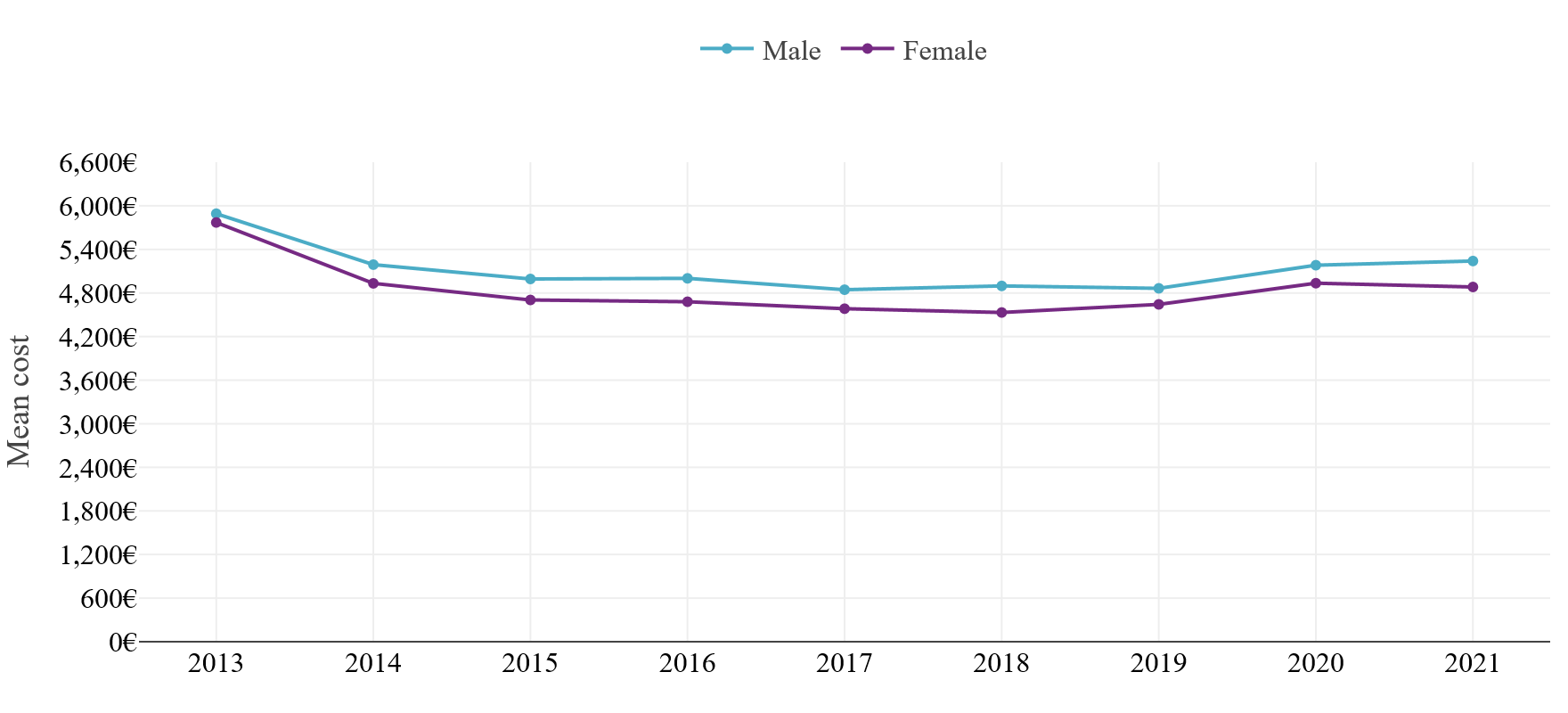


Suppl Fig 1: Mean monthly costs per patient living with metastatic lung cancer between 2013 and 2021, by sex


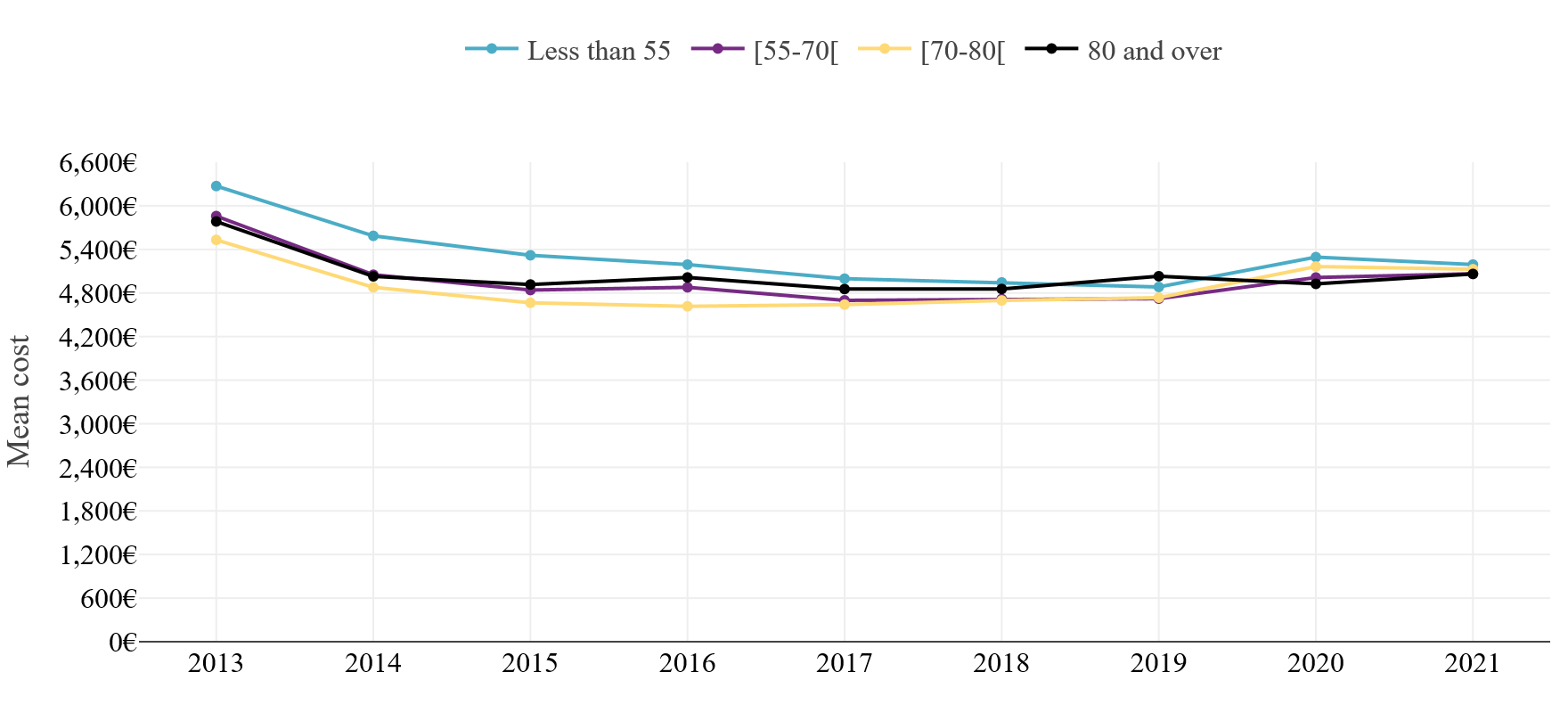


Suppl Fig 2: Mean monthly costs per patient living with metastatic lung cancer between 2013 and 2021, by age subgroup
